# Using Digital Image Correlation on SEM Images of Strain Field after Ion Beam Milling for the Residual Stress Measurement of Thin Films

**DOI:** 10.3390/ma13061291

**Published:** 2020-03-12

**Authors:** Terry Yuan-Fang Chen, Yun-Chia Chou, Zhao-Ying Wang, Wen-Yen Lin, Ming-Tzer Lin

**Affiliations:** 1 Department of Mechanical Engineering, National Cheng Kung University, Tainan 701, Taiwan; ctyf@mail.ncku.edu; 2 Graduate Institute of Precision Engineering, National Chung Hsing University, Taichung 402, Taiwan; bj611221@gmail.com (Y.-C.C.); g2001ya@yahoo.com.tw (Z.-Y.W.); willylin710@gmail.com (W.-Y.L.); 3 Research Center for Sustainable Energy and Nanotechnology, National Chung Hsing University, Taichung 402, Taiwan

**Keywords:** residual stress, ring-core drilling, digital image correlation (DIC)

## Abstract

The residual stress of thin films during the deposition process can cause the components to have unpredictable deformation and damage, which could affect the service life and reliability of the microsystems. Developing an accurate and reliable method for measuring the residual stress of thin films at the micrometer and nanometer scale is a great challenge. To analyze the residual stress regarding factors such as the mechanical anisotropy and preferred orientation of the materials, information related to the in-depth lattice strain function is required when calculating the depth profiles of the residual strain. For depth-resolved measurements of residual stress, it is strategically advantageous to develop a measurement procedure that is microstructurally independent. Here, by performing an incremental focused ion beam (FIB) ring-core drilling experiment with various depth steps, the digital image correlation (DIC) of the specimen images was obtained. The feasibility of DIC to FIB images was evaluated after the translation test, and an appropriate procedure for reliable results was established. Furthermore, the condition of the film in the function of residual stress was assessed and compared to elucidate the applicability of this technology.

## 1. Introduction

For modern microsystem technologies, it is important to understand the residual stress of thin films, as they might cause some unforeseeable damages or deformation to the components because of the residual stress accumulated during the deposition. It is a great challenge to conduct a reliable modeling and measurement for the residual stress of micro and nanostructured thin films. In order to realize the residual stress in a manner that considers the mechanical anisotropy and the preferred orientation of the materials, the detailed understanding of strain function corresponding to the depth lattice is required when analyzing depth profiles of residual strain [1].

At present, there are several ways to measure residual stress, which can be divided into non-destructive, semi-destructive, and destructive. Among them, the most commonly used is the non-destructive measurement method, particularly the X-ray diffraction (XRD). The advantage of the XRD method is that it is surface-sensitive and phase selectable. However, the lattice function of the material must be considered, and it cannot measure amorphous materials. Therefore, developing a method for the depth-resolving measurement for the residual stress of the film that is independent of microstructure has become a high-profile issue [2,3].

Recently, it is possible to obtain the full-field deformation behavior of thin film materials by using digital image correlation (DIC) on scanning electron microscopy (SEM) images [2]. Sutton [4,5,6] originally introduced the DIC techniques that calculated the grayscale digital image of the object translation through bilinear interpolation. It was advised that a continuous smooth secondary function can be optimized by the interpolation of a discrete digital image. The grayscale information was used from an integer pixel to a sub-pixel [7]. Later, in order to solve the six unknown varieties, he illustrated the use of the Newton–Raphson method, which was setting an initial guess value before the iteration method. This method has solutions that convert appropriate range and allow variables to be found more efficiently [6]. Recently, there is a new method to rebuild the image, which is named bi-cubic spline interpolation. The usage of bi-cubic spline interpolation was found to be more accurate than that of bilinear interpolation [7]. Here, by combining digital image correlation (DIC) techniques and focus ion beam (FIB) [2,3], it is possible to measure the residual stress through the independent procedure of ring core drilling for the resolving depth method without any knowledge of the crystallographic and phase structure of the thin films. Korsunsky et al. [8] and the following [9,10] opened up to report the state of the art in depth profiling by FIB-DIC micro-ring-core (equi-biaxial and general). Later, Lord et al. [11], Archie et al. [12], and Salvati et al. [10] provided very good practice guidelines for FIB-DIC measurements for micro-nanoscale thin films. Recently, Korsunsky et al. [13], Renzelli et al. [14], Bemporad et al. [1], and Siavash Maraghechi et al. [15] illustrated the standard practice guidelines for FIB-DIC measurements for thin films and coatings with different fabrication processes and tailored residual stress profiles. Each of these papers has its merits and provides very useful information in studies of the applicability of the DIC technique and image correlation and related measurement errors. In general practice, the development of best practices for SEM image acquisition for DIC application may face many complex problems. In particular, it is not easy to correctly implement these images when DIC is implemented. One of the major problems is the application of a reliable pattern on the sample surface for the tracking of one image to another. Kammers and Daly [16] illustrated the DIC methods of SEM images and noted that more complex issues may be encountered by using limited SEM surface image patterns and pixels to correctly implement these techniques. Despite a high-quality pattern that can be artificially utilized for a better observation, the mechanical behavior of sample structures may be influenced because of the patterning procedure. The high magnification of SEM introduces the complex image distortions. The long scanning time for each image captures high-sensitivity noises; moreover, it will drift from SEM parameters. As a result, an appropriate development scheme for the digital correlation of SEM images with natural surface features is essential. Siavash Maraghechi et al. [17] pointed out that the SEM images may have a significant amount of imaging artefacts. This situation could generate localization distortions in the displacement fields obtained from the images analyzed by DIC [17]. Despite many research studies on the DIC of SEM [1,2,4,13,14,15,16], the computational accuracy and the speed of advancement are always the major subjects. Prior to this, a study was performed on the validation methodology of DIC for SEM to reduce errors and use it for strain and surface deformation measurement of Complementary Metal-Oxide-Semiconductor Integrated Circuit (CMOS IC) samples. This DIC method and its parameters on SEM images were validated for testing under spatial translation or time [18]. In the study, the stability, accuracy, and speed of calculation were compared by utilizing various image correlation criteria; the accuracy and stability of Zero-Normalized Sum of Squared Differences (ZNSSD) and Sum of Squared Differences (SSD) of DIC were studied. The results of applications in real SEM images of ICs were guided for the standard choice of analysis parameters in application of the full-field displacement measurements.

In this study, the digital image correlation method with the appropriate image correlation criteria suggested in the prior study using ZNSSD was performed to acquire more stable and accurate specimen images. By using incremental focused ion beam (FIB) ring-core drilling with different depth steps, the images were captured and correlated. The FIB-DIC method allows calculating the full 2D displacement field within the gauge area and the surface strain obtained from the digital image correlation method on SEM images. Then, using the proper form of Hook’s law on the measured displacement field leads to the evaluation of the residual stress field of thin films. The study used a ring core drilling measurement method, which can be applied to crystalline and amorphous materials. Further, in order to measure the relaxation strain when the material was milled, the semi-destructive measurement methods were performed.

## 2. Method

**Figure 1:**
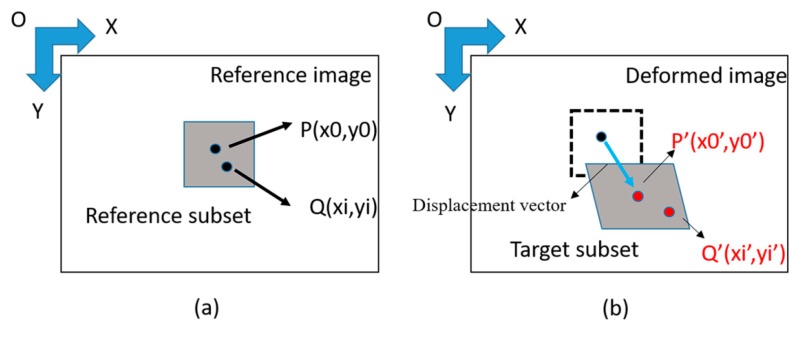
The position of the object (**a**) before deformation (**b**) after deformation [18].

In the DIC method, the constraints of the sample were modified by the milling process disrupting the equilibrium state and leading to deformations within the gauge volume. It was assumed that the feature points on the surface of the object were consistent with the deformation of the object; therefore, the relative position of the feature points on the surface of the object had been analyzed by the image before and after the deformation, and the amount of deformation was obtained. The center point of a subset on the surface of the object is P (x_0_, y_0_), and a point inside the subset is Q (x, y). When the object was deformed, the new coordinate of this point is (x′, y′); such was the relative position before and after the deformation, as indicated in Figure 1. The x′ and y′ can be expressed as:

x′ = x + u(x, y)(1)

y′ = y + v(x, y)(2)

where u (x, y) is the horizontal displacement of the object deformation, and v (x, y) is the vertical displacement of the object deformation.

Previously, the measurement stability, accuracy, and speed of calculation using different image correlation criteria of DIC on SEM images were investigated [18]. The accuracy and stability of ZNSSD and the Sum of Squared Differences (SSD) of DIC were compared. The results were used to measure the accurate full-field displacement here. For the first SEM image, the full fields in the region are all at 0. This image was compared with the SEM image of the sample after deformation. The full-field displacements of each image analyzed by DIC are the difference between the whole image and the first surface SEM displacement. The DIC images we used in this study have the scale of 120 pixels per micron, which is 120 pixel/μm of the displacement fields.

For the FIB-DIC, the principle of drilling is mainly based on the change in the shape of the object caused by the release of stress in the ring core. The strain of the subject released after drilling and the surface residual stress were calculated using the theory of elasticity.

Based on the principle of fundamental mechanics, the stress components perpendicular to the Z direction of the surface, such as *σ*_xz_, *σ*_yz_, and *σ*_zz_ are all zero; therefore, the stress on the surface at the time of drilling is in equilibrium, which must be redistributed and released.

**Figure 2:**
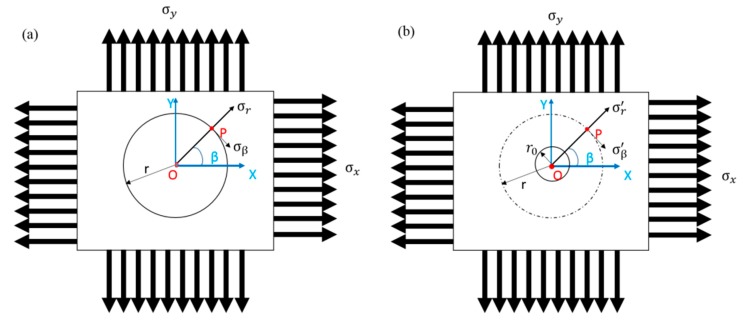
Infinite flat plate subjected to a biaxial tensile stress diagram (**a**) before drilling and (**b**) after drilling.

If a circular core with a radius $r_{0}$ is subjected to a uniaxial plane tensile stress *σ*_x_ on the plate, as shown in Figure 2, the stress state around the circular core can be divided into the superposition of Figure 2a,b.

By substituting the known three angles and the three strain values in Figure 2, the stress state of P (r, β) before milling can be calculated using the following [3,19]:

(3) $$\sigma_{r}=\frac{\sigma_{x}}{2}\;(1+\cos2\beta )$$

(4) $$\sigma_{\beta }=\frac{\sigma_{x}}{2}\;(1-\cos2\beta )$$

(5) $$\sigma_{\beta }=\frac{\sigma_{x}}{2}\;(1-\cos2\beta )$$

where $\sigma_{r}$ is the stress component along the r direction before milling, $\sigma_{\beta }$ is the stress component perpendicular to the r direction, $\sigma_{x}$ is a uniaxial plane tensile stress, $\tau_{r\beta }$ is the shear stress, and β is the angle between position vector P and $\sigma_{x}$ direction.

The stress of the plane does not only include a single axial stress, but other stress in the same plane and another axial stress was also considered, as shown in Figure 2. Under the theory of elasticity, the principle of superposition can be applied to the stress state, which is expressed as:

*σ*_β_′ = *σ*_β_ + Δ*σ*_β_. (6)

The residual stress components *σ*_γ′_ and *σ*_β′_ at the point P (r, β) are expressed as:

(7) $$\sigma_{\gamma }^{\prime }=\frac{\sigma_{x}}{2}[1-{\left(\frac{\gamma_{0}}{\gamma }\right)}^{2}+\frac{\sigma_{x}}{2}[1-4{\left(\frac{\gamma_{0}}{\gamma }\right)}^{2}+3{\left(\frac{\gamma_{0}}{\gamma }\right)}^{4}]\cos2\beta +\frac{\sigma_{y}}{2}[1-{\left(\frac{\gamma_{0}}{\gamma }\right)}^{2}]-\frac{\sigma_{y}}{2}[1-4{\left(\frac{\gamma_{0}}{\gamma }\right)}^{2}+3\left(\frac{\gamma_{0}}{\gamma }{)}^{4}\right]\cos2\beta$$

(8) $$\sigma_{\beta }^{\prime }=\frac{\sigma_{x}}{2}[1-{\left(\frac{\gamma_{0}}{\gamma }\right)}^{2}]+\frac{\sigma_{x}}{2}[1+3{\left(\frac{\gamma_{0}}{\gamma }\right)}^{4}]\cos2\beta +\frac{\sigma_{y}}{2}[1-{\left(\frac{\gamma_{0}}{\gamma }\right)}^{2}]+\frac{\sigma_{y}}{2}[1+3\left(\frac{\gamma_{0}}{\gamma }{)}^{4}\right]\cos2\beta$$

where $\gamma_{0}$ is the drilling radius, γ  is the radius distance from the core center, and $\sigma_{y}$ is a uniaxial plane tensile stress.

The relationship between the stress components *σ*_γ_ and *σ*_β_ at point P (r, β) before drilling and the plane principal stresses *σ*_x_ and *σ*_y_ are expressed as:

(9) $$\sigma_{\gamma }=\frac{\sigma_{x}+\sigma_{y}}{2}+\frac{\sigma_{x}-\sigma_{y}}{2}\cos2\beta$$

(10) $$\sigma_{\beta }=\frac{\sigma_{x}+\sigma_{y}}{2}-\frac{\sigma_{x}-\sigma_{y}}{2}\cos2\beta .$$

Before and after drilling, the amount of stress changed, and the amount of stress released at P (r, β) was calculated using:

(11) $$\Delta \sigma_{\gamma }=-\frac{\sigma_{x}+\sigma_{y}}{2}{\left(\frac{\gamma_{0}}{\gamma }\right)}^{2}+\frac{\sigma_{x}-\sigma_{y}}{2}(-4{\left(\frac{\gamma_{0}}{\gamma }\right)}^{2}+3\left(\frac{\gamma_{0}}{\gamma }{)}^{4}\right)\cos2\beta$$

(12) $$\Delta \sigma_{\beta }=\frac{\sigma_{x}+\sigma_{y}}{2}{\left(\frac{\gamma_{0}}{\gamma }\right)}^{2}-\frac{\sigma_{x}-\sigma_{y}}{2}[3\left(\frac{\gamma_{0}}{\gamma }{)}^{4}\right]\cos2\beta .$$

According to Kabiri’s [3], the radial strain Δε_γ_ was measured because the radial strain change is much larger than the circumferential strain change Δε_β_, so the circumferential strain can be neglected; therefore, the biaxial Hooke’s Law for stress–strain conversion was used in this study, which is expressed as:

(13) $$\Delta \varepsilon_{r}=(A+\mathrm{Bcos}2\beta )\;\sigma_{x}+(A-\mathrm{Bcos}2\beta )\;\;\sigma_{y}$$

where ν is the Poisson’s ratio, $\gamma_{0}$ is the drilling radius, γ  is the radius distance from the core center, A is the correction coefficient, $\frac{1+\nu }{8E}$($\frac{2r_{0}}{r}{)}^{2}$, and B is the correction coefficient, $\frac{1}{E}$[$\frac{1}{2}$($\frac{2r_{0}}{r}{)}^{2}$ − $\frac{3\left(1+\nu \right)}{32}$($\frac{2r_{0}}{r}{)}^{4}$].

Based on the above equation, three different directions of strain values are required. Next, the distances *r* and β = 0°, β + φ = 45°, β +∅ = 90°, and the points P (*r*, *β*), Q (*r*, *β* + *φ*)**,** R (*r*, *β* + ∅) were used to obtain the strain, which are expressed as:

(Δ*ε*_γ_)_P_ = (*A* + *B*cos2*β*)*σ*_x_ + (*A* − *B*cos2*β*)*σ*_y_ (14)

(Δ*ε*_γ_)_Q_ = [*A* + *B*cos2(*β* + *φ*)]*σ*_x_ + [*A* − *B*cos 2(*β* + *φ*)]*σ*_y_ (15)

(Δ*ε*_γ_)_R_ = [*A* + *B*cos2(*β* + ∅)]*σ*_x_ + [*A* − *B*cos 2(*β* + ∅)]*σ*_y._ (16)

If ∅ = $\frac{\pi }{4}$, *φ* = $\frac{\;\pi }{2}$, the simultaneous equations can be expressed as:

(17) $$\sigma_{x}=\frac{\varepsilon_{p}+\varepsilon_{R}}{4A}+\frac{\sqrt{2}}{4B}\sqrt{[\varepsilon_{p}-\varepsilon_{Q}{]}^{2}+[\varepsilon_{Q}\;-\;\varepsilon_{R}{]}^{2}}$$

(18) $$\sigma_{y}=\frac{\varepsilon_{p}+\varepsilon_{R}}{4A}-\frac{\sqrt{2}}{4B}\sqrt{[\varepsilon_{p}-\varepsilon_{Q}{]}^{2}+[\varepsilon_{Q}\;-\;\varepsilon_{R}{]}^{2}}$$

(19) $$\beta =\frac{1}{2}tan^{-1}\frac{{\left(\Delta \varepsilon_{\gamma }\right)}_{P}-2{\left(\Delta \varepsilon_{\gamma }\right)}_{Q}+{\left(\Delta \varepsilon_{\gamma }\right)}_{R}}{{\left(\Delta \varepsilon_{\gamma }\right)}_{P}-{\left(\Delta \varepsilon_{\gamma }\right)}_{R}}.$$

For the strain, the three strain values, P, Q, and R were measured at the three different angles denoted as β, β + φ, and β + ∅:

(20) $$\varepsilon_{P}=\frac{\varepsilon_{x}+\varepsilon_{y}}{2}+\frac{\varepsilon_{x}-\varepsilon_{y}}{2}\;\cos2\beta +\frac{1}{2}\gamma_{\mathrm{xy}}\sin2\beta$$

(21) $$\varepsilon_{Q}\;=\frac{\varepsilon_{x}+\varepsilon_{y}}{2}+\;\frac{\varepsilon_{x}-\varepsilon_{y}}{2}\;\cos2(\beta +\varphi )+\frac{1}{2}\gamma_{\mathrm{xy}}\sin2(\beta +\varphi )$$

(22) $$\varepsilon_{R}=\frac{\varepsilon_{x}+\varepsilon_{y}}{2}+\frac{\varepsilon_{x}-\varepsilon_{y}}{2}\;\cos2(\beta +\text{∅})+\frac{1}{2}\gamma_{\mathrm{xy}}\sin2(\beta +\text{∅})$$

where $\varepsilon_{P}\;$ is the strain value at the angle β on the *x*-axis, $\varepsilon_{Q}\;$ is the strain value at the angle (β + φ), and $\varepsilon_{R}\;$ is the strain value at the angle (β + ∅).

As a result, the relaxation strain and residual stress after each milling can be obtained.

## 3. Experimental Procedure

**Figure 3:**
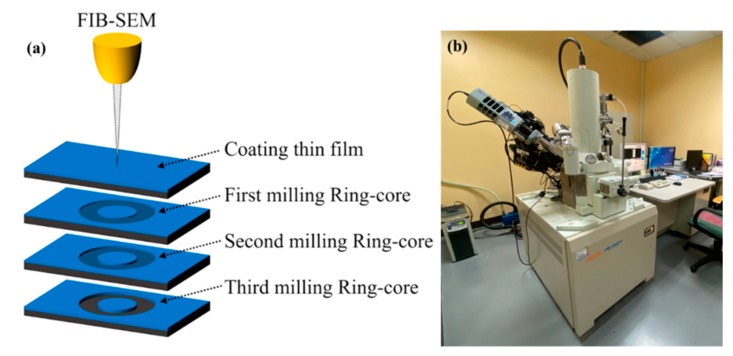
(**a**) Focused ion beam (FIB)-SEM process diagram. (**b**) FIB-SEM experimental system (Equipment JEOL JIB-4601F).

In order to demonstrate the feasibility of the method, two kinds of thin films including a metal and transition metal compound were tested. Ag was used as an example because it is a noble metal and has consistent properties after deposition. ZrN was used as a hard coating compound sample in contrast of metals. Both films were sputtered deposited and analyzed in this work. The sputtering parameters are shown in Table 1 and Table 2. During the experiment, a designated thin film layer deposited on a silicon substrate was used as a test piece. The FIB was used to carry out the milling process of the ring-core. The scanning electron microscope was used to capture the milled image, and the displacement and strain of the samples were analyzed by the DIC method, as illustrated in Figure 3.

The specimen was placed horizontally into the FIB instrument (JEOL, Tokyo, Japan) to have a vertical relationship with the SEM (JEOL, Tokyo, Japan) electron gun, as shown in Figure 3. For ion beam milling, the specimen was placed perpendicular to the ion beam gun so that the specimen carrier could be milled perpendicular to the ion beam gun. The image resolution, brightness, contrast, magnification, and surface image range correction were adjusted throughout sensitivity tests so that the image can be clearly displayed and can be shot after the next FIB ion beam milling. The ion beam parameters adjusted include the beam, the dose, and the size of the ring to be milled. The beam represents the beam current (pA) of the ion beam. The current level was set at 1–13, as shown in Table 3. The smaller the number, the larger the current of the ion beam. It is true that the beam also affects the milling time and the flatness of the cut ring. In this study, the beam level used in the experiment was 7 for the Ag film and 10 for the ZrN film after preliminary optimization studies. The beam grade is the current energy (pA) of the ion beam, grades 1 to 13. The amount of energy is related to the flatness of the edge of the milled pattern. The smaller the value, the greater the energy. Here, Ag and ZrN have obtained the best parameter solution related to time and quality through experiments. The dose represents the current per unit area of the ion beam (nC/μm^2^). It refers to the time it takes to reach the dose. The higher the number, the longer the time it takes. The optimized dose used in this study was a minimum of 0.1 (nC/μm^2^).

Ion beam milling of different shapes and sizes was set according to the needs of the experiment. In this study, the shape of the circular ring used with D_0_ (3 μm) as the outer diameter of the ring and D (1.5 μm) as the inner diameter of the ring.

For the SEM image and the ion beam parameters, the range in the SEM image was first set. Here, both the SEM of Ag and ZrN thin film were set up at a magnification of 12,000×. Next, the SEM surface image was taken, which served as the benchmark for subsequent DIC image analysis, before the milling of the ring was started. Then, the FIB milling parameters were set to start the milling of the ring on the test piece. After the milling operation, a second SEM surface image was taken, and the shift of the first ring milling was taken after the shooting. After calibration, the second ring milling was done, and the third SEM surface ring image was taken. This procedure was repeated until the film was drilled to the bottom of the film layer. A total of four millings were done and five SEM milling ring images were taken (see Figure 3). Finally, SEM images were analyzed by DIC.

Since each stage of FIB milling obtained a slight shift during SEM electron gun image shooting, each SEM image of FIB milling had a slight difference in position with the first SEM image of the FIB milling surface. Therefore, an area in the first surface SEM image without FIB milling was selected—specifically, the area in the next FIB milling ring that was not affected by the deformation, for calibration. The same area on the SEM image was framed as the first surface SEM image, and the first frame selection area was used as the reference.

**Figure 4:**
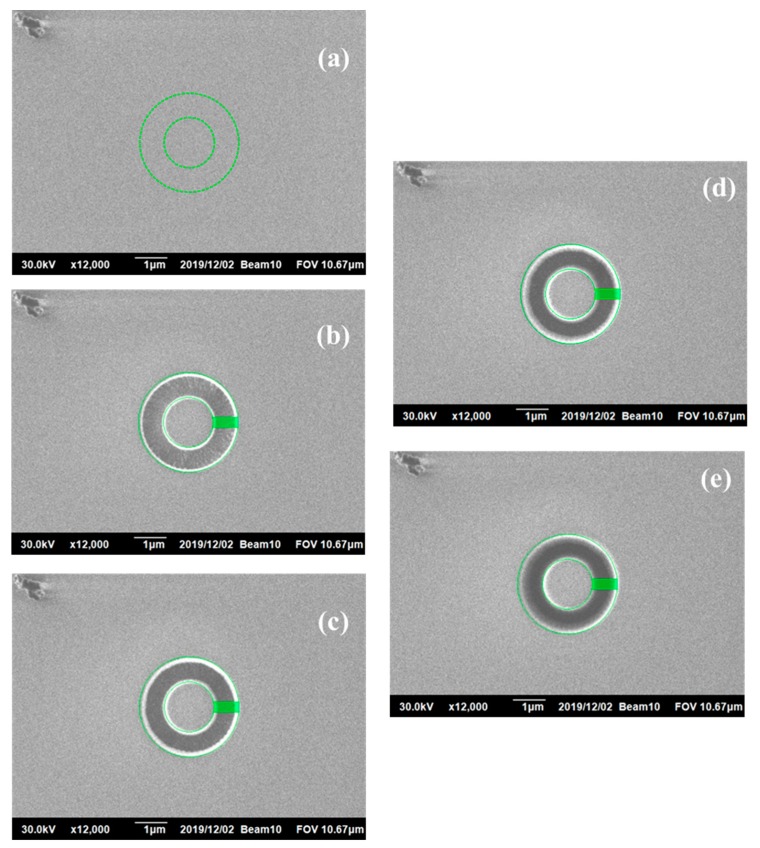
FIB-DIC (digital image correlation) images of ZrN thin film at a magnification of 12,000×. Milling depth to surface: (**a**) positioned simple before milling, 0 μm; (**b**) first milling depth, 0.6 μm; (**c**) second milling depth, 0.8 μm; (**d**) third milling depth, 1.0 μm; (**e**) fourth milling depth, 1.1 μm.

An example on the surface images of sputtered ZrN thin films deformed throughout each step of FIB milling is presented in Figure 4. Figure 4a shows the SEM image before the milling. After the drilling, the DIC images for each step were taken by SEM as shown in Figure 4b–e. The nature surface features were directly used for image correlation. The drift distortion, noises, and time-dependent nature of the SEM images, and the electromagnetic lens effects, were all considered. DIC codes were programed on the SEM images of FIB samples captured. Inside the images, multiple matrices of the unit cubic consisting of 5 *×* 5 pixels were used for the subset and with the corresponding interval of an additional 3 pixels’ distance to their overlap sizes after deformation to calculate the correlation using a grayscale digital image through ZNSSD bilinear interpolation for the object translation. The fitting tool (cftool function of Matlab) was used for DIC post-processing to obtain the full field displacement graph and strain field. 

The residual stress was calculated using Matlab routine codes based on the equations illustrated in the Method section. Compared to traditional measurements, the FIB combined with SEM image observation achieved accurate sub-micron measurements, allowing the milling shape to be adjusted according to the requirements, and greatly shortening the experimental analysis time during the rapid measurement of DIC.

## 4. Results and Discussion

### 4.1. DIC Displacement Measurement

**Figure 5:**
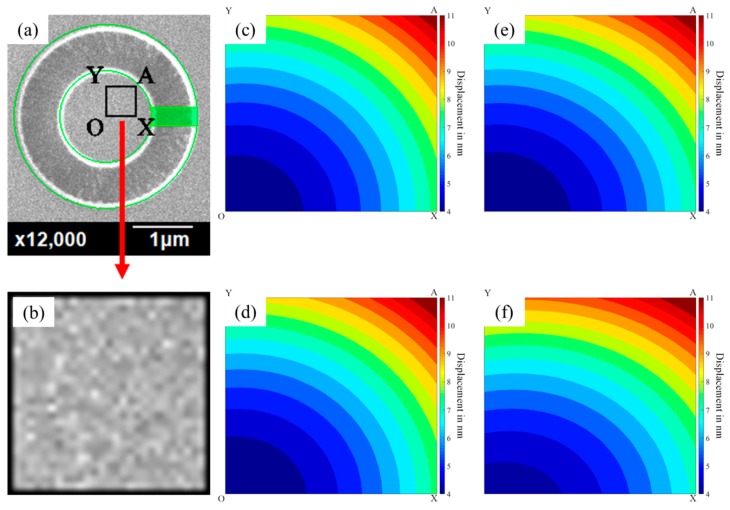
The deformation image of FIB-DIC was analyzed through the displacement distribution of the field on the ZrN thin film at a magnification of 12,000× by DIC performed in Figure 4. As a result of the symmetrical deformation pattern of the ring core, these results only present the first quarter of the ring with the rectangular area of point O, X, A & Y. As illustrated: (**a**) the whole SEM image of the sample surface taking after milling; (**b**) Zooming SEM image with the rectangular area of point O, X, A & Y; (**c**) Displacement field of the rectangular area of point O, X, A & Y after the first milling depth, 0.6 μm; (**d**) Displacement field of the rectangular area of point O, X, A & Y after the second milling depth, 0.8 μm; (**e**) Displacement field of the rectangular area of point O, X, A & Y after the third milling depth, 1.0 μm; (**f**) Displacement field of the rectangular area of point O, X, A & Y after the fourth milling depth, 1.1 μm.

Full-field measurements of the surface deformation of each sample on each milling step were performed by DIC. The selected deformation area must be measured before starting the DIC; then, it uses the 15,500 pixel data in this area to begin the observation, as shown in Figure 5. An example of the surface deformation measurement results on the ZrN thin film performed in Figure 4 after four milling steps from 0.6 to 1.1 μm depth from the surface are shown in Figure 5.

As shown in Figure 5, the deformation image of FIB-DIC was analyzed through the displacement distribution of the field on the ZrN thin film at a magnification of 12,000× by DIC performed in Figure 4. As a result of the symmetrical deformation pattern of the ring core, these results only present the first quarter of the ring with the rectangular area of point O, X, A & Y in Figure 5a. As illustrated, these include: (a) the whole SEM image of the sample surface taking after milling; (b) Zooming SEM image with the rectangular area of point O, X, A & Y; (c) Displacement field of the rectangular area of point O, X, A & Y after first milling depth, 0.6 μm; (d) Displacement field of the rectangular area of point O, X, A & Y after second milling depth, 0.8 μm; (e) Displacement field of the rectangular area of point O, X, A & Y after third milling depth, 1.0 μm; and the (f) Displacement field of the rectangular area of point O, X, A & Y after fourth milling depth, 1.1 μm. In this figure, the dark red area represents a maximum displacement of 11 nm, and the blue area represents a minimum displacement of 4 nm.

As illustrated in Figure 5, the displacements near the center are more uniform. However, due to different material hardness, the surface will be out-of-flatness after FIB milling. To calculate the residual stress more accurately, choosing the more flat area that is near the center and avoiding the uneven area were very important. However, the selected area might be affected significantly during the next milling. Based on this reason, not only the even color but also the COR (correlation coefficient) value would be considered.

**Figure 6:**
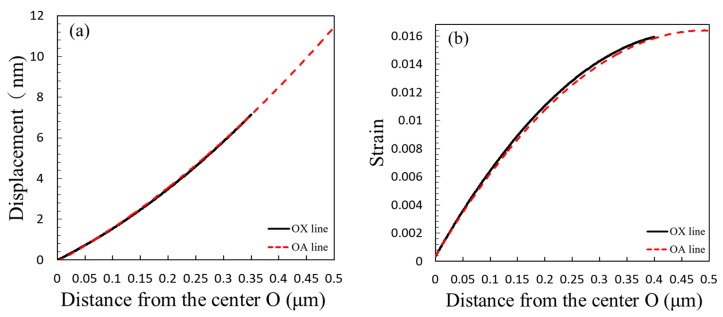
The illustration on an example of the displacement (**a**) and strain (**b**) along the rectangle coordinate of the edges OX and OA line from the center O of the ring core on Figure 5c.

Following the surface deformation measurement, the *ε*_x_, *ε*_y_, and *γ*_xy_ strain distributions at different milling depths could therefore be obtained by DIC analysis. The strain fields were using a routine to differentiate the displacement fields. An example of the displacement and strain along the rectangle coordinate of the edges on Figure 5c is shown in Figure 6. This strain on the ZrN thin film was after one milling step of 0.6 μm to 0.8 μm depth from the surface. The region with uniform strain distribution was selected, and the angles 0°, 45°, and 90° corresponding to the OX, OA, and OY lines were defined on the surface of the ring, respectively. The three coordinate points on the three angular axes *ε*_x_, *ε*_y_, and *γ*_xy_ were used in the formula to calculate the coordinate points ε_P_, ε_Q_, and ε_R_. Then, the residual stress formula was used to obtain the residual stress value from the surface of the ring.

### 4.2. Residual Stress of Ag Thin Films

**Figure 7:**
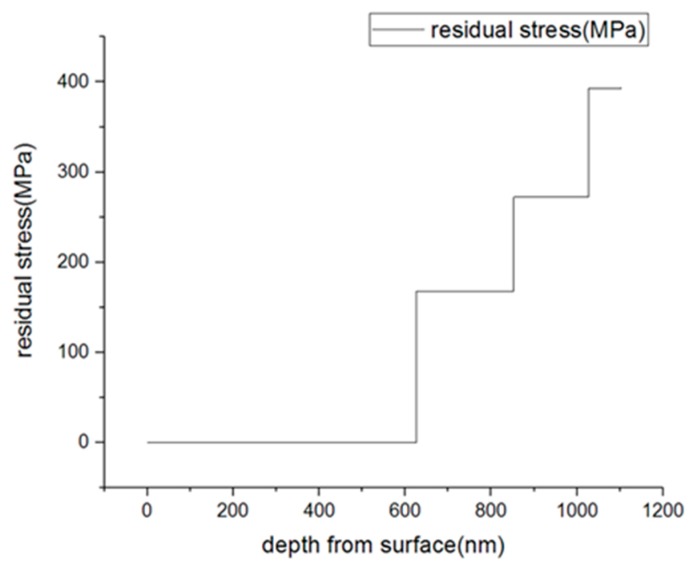
Relaxed residual stress measurement of an Ag thin film of FIB-DIC.

The results of the residual stress on an Ag thin film are shown in Figure 7. The results of the residual stress plotted were represented as a mean value calculated from the inside perimeter of the linear strain field of the ring-core. As shown in Figure 7, the relaxation residual stress was found to be 167.45 MPa at the milling depth of 0.6 μm from the surface. The relaxation stress increases as the milling depth increases. It rises to 272.01 MPa as the milling depth declines down to 0.8 μm from the surface. The stress continuous to increase and reaches 392.35 MPa when the milling depth reaches 1.0 μm and lastly, it reaches 393.66 MPa when the milling depth reaches 1.1 μm from the surface.

**Figure 8:**
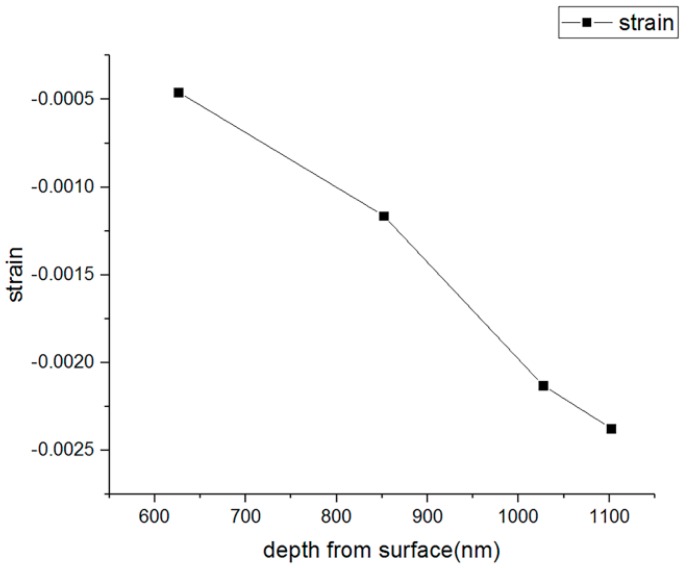
Strain at different depths.

Figure 7 shows a plot of the relaxed residual stress versus milling depth, which indicates that the residual stress on this particular Ag thin film was around 150 to 450 MPa from top to bottom. Figure 8 shows a plot of the relaxation strain versus milling depth. The negative strains indicate the presence of a tensile stress inside the film. For the Ag thin films, it is clear from both plots that there was an increase in the stress state as the milling depth increased from the surface to the interface. The results were compared to the values obtained from the other literature, and these are summarized in Table 4. 

### 4.3. Residual Stress of ZrN Thin Films

**Figure 9:**
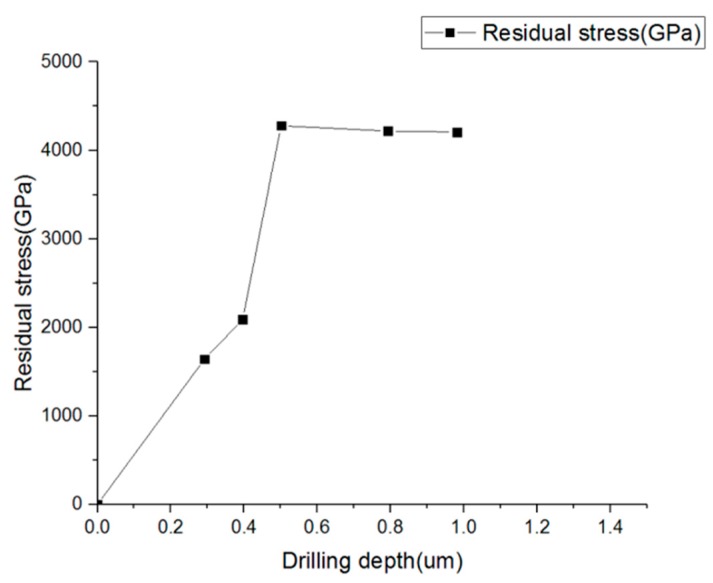
Relaxed residual stress measurement of ZrN of FIB-DIC.

The results of the residual stress on a ZrN thin film are shown in Figure 9. As shown in Figure 9, the relaxation residual stress was found to be 1640.6 MPa at the milling depth of 0.3 μm from the surface. The relaxation stress increases as the milling depth increases before it reaches the half depth. It rises to 2087.7 MPa as the milling depth declines down to 0.4 μm from the surface. The stress continuous to increase and reaches 4279.8 MPa when the milling depth reaches 0.5 μm. It seems that the relaxation residual stress reaches a plateau after passing half the depth of the surface, and the residual stress was 4219.0 MPa with the milling depth of 0.7 μm and 4208.3 MPa with the milling depth of 1 μm. The results were compared with the values obtained from other literature and are summarized in Table 5. The stress values and Table 5 include those obtained from XRD results, which are consistent with FIB-DIC values.

The stress values and Table 5 include those obtained from XRD, and the curvature measurement results are found to be consistent. According to the literature listed in Table 5, the residual stress increases as the depth increases. The trend of the depth measurement from 600 to 1000 nm in the literature was fit and compared with our result of the depth measurement from the surface to the bottom in Figure 9.

Figure 9 clearly shows that the average stress obtained by the FIB-DIC ring-core method is methodically higher than those obtained by XRD results shown in Table 5. A recent study of the residual stress measurement of ZrN coating thin films by Bemporad et al. [11] indicated that the difference between XRD is in the order of 20% when it considers the (111) XRD measurement result, and it is possibly greater if the (200) reflection is used to compare with FIB-DIC. They explain this because of the differences between the volumes of material sampled by the two methods, while the gauge volume in the FIB-DIC method is of the order of 10 μm^3^ and the penetration depth is equal to the coating thickness, but the X-ray diffraction setup probes primarily the surface layers. Therefore, the near-surface regions have less compression compared with the deeper area. Therefore, the stress values obtained from XRD analysis show less compression stress than those measured by this method that accesses a greater proportion of the film thickness.

In general, ZrN is a very hard coating thin film with relatively significant high residual stress. The stress depth profiles indicate a stress gradient of stress through the film thickness. In particular, the stress gradient is much steeper near the surface residual stress value compared to that at half the depth of the thickness. The residual stress reaches a plateau after passing the half depth of the surface and remains consistent through to the bottom, which indicates that the relaxation stress of the hard coating releases significantly from the beginning of the drill. As a result, the FIB-DIC method can also provide an accuracy of estimated value of the very near-surface residual stress, which is not feasible from any other methods for the thin film residual stress measurement.

An internal analysis of the ring-core has been performed. The residual stress information provided from the ring-core shows local stress information. However, the integration of multiple ring-cores tested in the sample could provide better understanding of the in-plane stress gradient which other methods cannot provide. Indeed, an external analysis of the ring-core could also be interesting for nanostructured thin films. Meanwhile, the integration of the multiple ring-cores tested in the sample could lead to understanding the external stress profiles, which is another point of interest.

## 5. Conclusions

This study performed a DIC method for the SEM images obtained through incremental ring-core drilling using FIB with various depth steps on both Ag and ZrN thin film samples. In order to study the applicability of DIC to FIB images, translation tests were calibrated initially, and then a proper procedure was performed to obtained accurate measurement results. Furthermore, the residual stress of Ag and ZrN thin film were studied and compared with XRD. The results showed that FIB-DIC has fewer restrictions on external conditions and material selection, and this method can also be performed for full-field analysis since the range required for the measurement and the range of applications is wider than that for other methods.

## Figures and Tables

**Table 1:** The sputtering deposition parameters of silver film.

Target	Ag (99.99%)
Background pressure (torr)	5 × 10^−6^
Working pressure (torr)	3 × 10^−3^
Argon gas flow (sccm)	13
Power (W)	115
Deposition rate (Å/sec)	6

**Table 2:** The deposition conditions of ZrN thin films.

Base pressure		5 × 10^−3^ Torr (6.7 × 10^−4^ Pa)
Deposition temperature		450 °C
Silicon substrate size		15 × 15 mm^2^
Bombardment	Bias	−600 V
	Working pressure	1.5 × 10^−3^ Torr
	Ar gas flow rate	30 sccm
	Bombarded time	5 min
Deposition conditions	Bias	−70 V
	Working pressure	1.5 × 10^−3^ Torr
	Ar gas flow rate	30 sccm
	N _2_ gas flow rate	1.3 sccm (0 sccm for Zr)
	Target current	0.37 A
	Hold rotation speed	18 rpm

**Table 3:** Ion beam current range for FIB.

Beam	1	2	3	4	5	6	7	8	9	10	11	12	13
**Ip (PA)**	60 K	30 K	10 K	3 K	1 K	500	300	100	50	30	10	3	1
**Aperture Size**	600	450	270	170	130	100	80	60	50	50	35	20	10

**Table 4:** The residual stress measurement results of Ag thin films compared with the values obtained from other literatures.

	50/50 nm Ag/Ni Bilayer [20]	7/7 nm Ag/Ni Multilayer [8]	1200 nm Ag Film [8]	400 nm Ag Film [9]
**XRD texture analysis (MPa)**	+130	+400	−	350
**Curvature method (MPa)**	−	−	310 ± 10	−

**Table 5:** The residual stress measurement results of ZrN thin films compared with the values obtained from the XRD of other literatures.

	1000 nm ZrN thin film [10]	300 nm ZrN thin film [21]	620 nm ZrN thin film [22]
**XRD texture analysis (GPa)**	−3.84 ± 0.09	−4.5	−3.73 and −4.01
